# Internal thoracic vein cannulation for venous port insertion

**DOI:** 10.1016/j.jvsv.2024.101887

**Published:** 2024-04-02

**Authors:** Ákos Bérczi, Péter Osztrogonácz, Csaba Csobay-Novák

**Affiliations:** a Department of Interventional Radiology, Semmelweis University, Budapest, Hungary; b Department of Vascular and Endovascular Surgery, Semmelweis University, Budapest, Hungary

**Keywords:** Chronic venous occlusion, Endovascular, Internal thoracic vein, Parenteral nutrition, Venous port

Chronic venous occlusion presents a significant challenge in patients requiring long-term venous access for parenteral nutrition.^1^, ^2^, ^3^ The placement and maintenance of venous port devices can also be difficult in such cases. The loss of central venous access in these patients is a complication that can lead to potential adverse events.^4^ We present a case that required parasternal insertion of a venous port via the internal thoracic vein in a 53-year-old female patient with Crohn disease. The patient provided written informed consent for the report of her case details and imaging studies.

**Figure:**
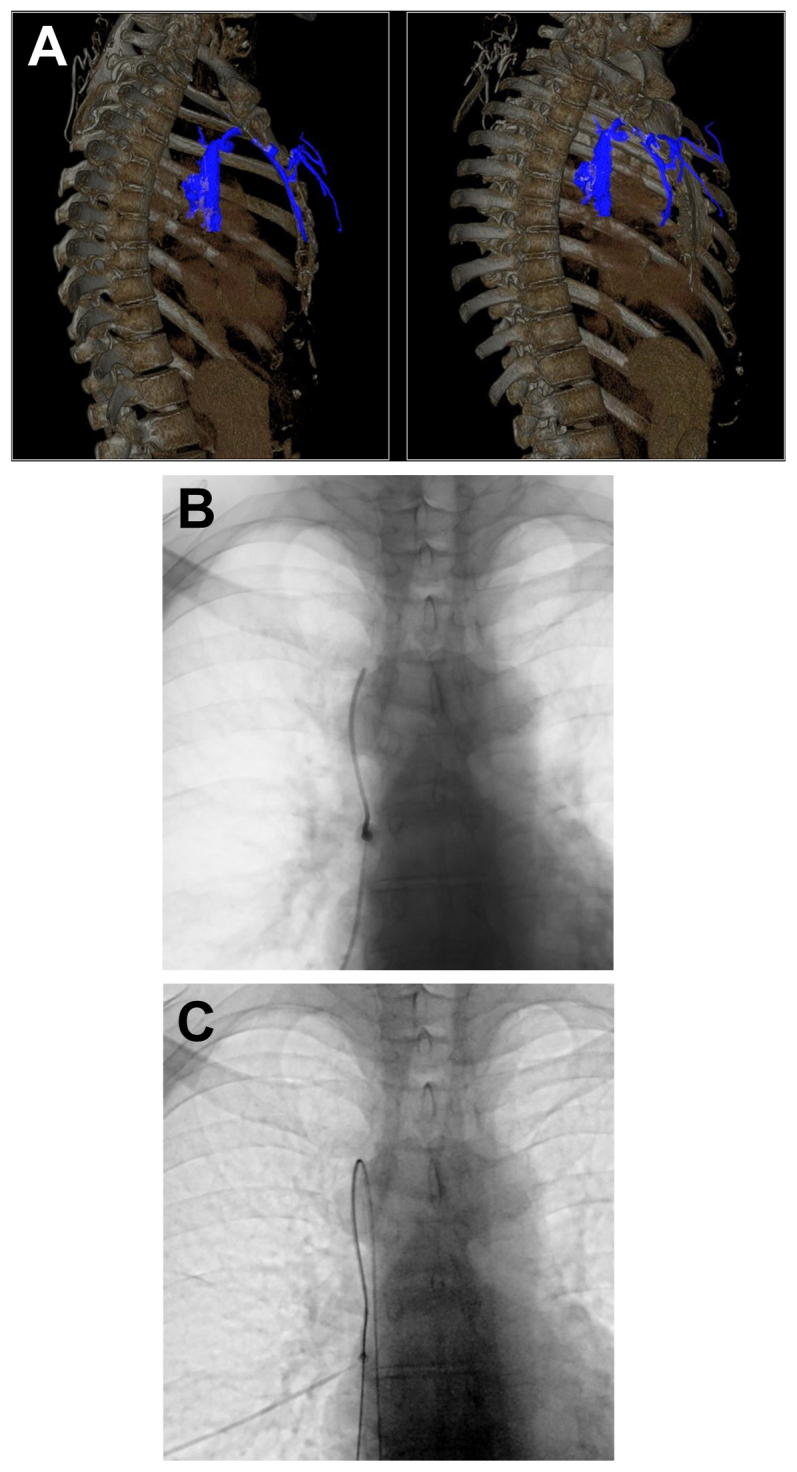

The initial computed tomography venography revealed chronic occlusions of the bilateral internal jugular and subclavian veins, which were caused by catheter-related thrombosis. Additionally, a large right internal thoracic vein with a diameter of 4 mm was detected (*A*/cover).

Ultrasound-guided puncture of the dilated superficial thoracic vein was performed under local anesthesia, followed by successful introduction of a 4F vascular access sheath (*B*). Fluoroscopic guidance and wire support were used to advance the catheter and position the tip in the right atrium (*C*).

Subsequently, a small incision on the anterior chest wall lateral to the access site established a 3- to 4-cm (about 1.5-in.) subcutaneous pouch. The catheter was trimmed to a suitable length and guided through a subcutaneous tunnel into the peel-away sheath. Finally, the port (Ambix Intraport; Fresenius Kabi Hong Kong Ltd) was placed in the subcutaneous pouch, and the wound was closed.

The internal thoracic vein could present a secure alternative for port placement in patients with challenging central venous access.

## Disclosures

None.

## Appendix

Cover Image
