# Supplementary figures and images for: Internal thoracic vein cannulation for venous port insertion
Source: J Vasc Surg Venous Lymphat Disord. 2024 Apr 2;13(1):101887. doi: 10.1016/j.jvsv.2024.101887 (PMC11764252; doi:10.1016/j.jvsv.2024.101887)

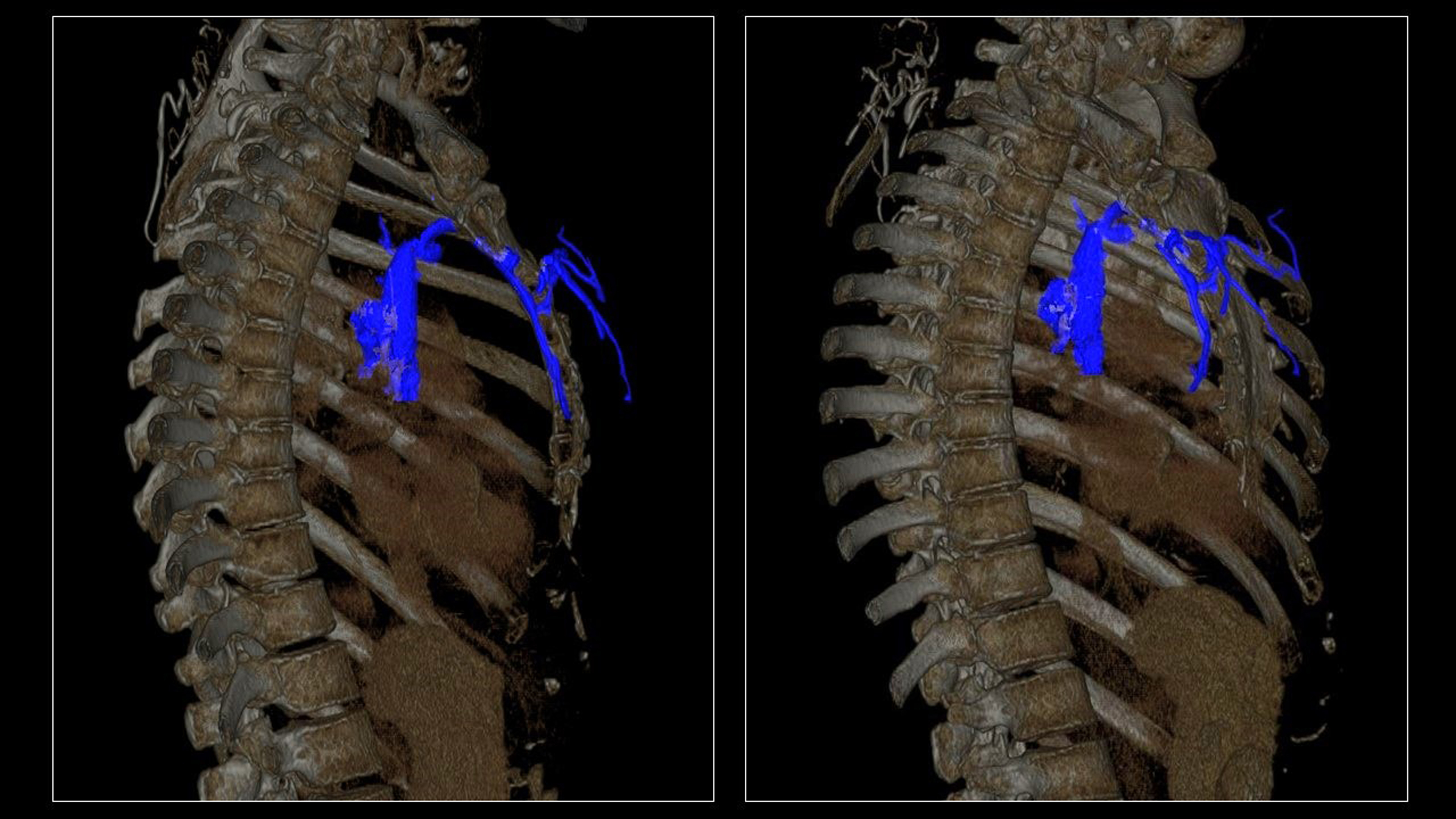

Supplement: Cover Image [file figs1.jpg]
